# Circulating Small Extracellular Vesicles Involved in Systemic Regulation Respond to RGC Degeneration in Glaucoma

**DOI:** 10.1002/advs.202309307

**Published:** 2024-06-25

**Authors:** Tong Li, Wen‐Meng Zhang, Jie Wang, Bai‐Jing Liu, Qiao Gao, Jing Zhang, Hai‐Dong Qian, Jun‐Yi Pan, Ming Liu, Qing Huang, Ai‐Wu Fang, Qi Zhang, Xian‐Hui Gong, Ren‐Zhe Cui, Yuan‐Bo Liang, Qin‐Kang Lu, Wen‐Can Wu, Zai‐Long Chi

**Affiliations:** ^1^ State Key Laboratory of Ophthalmology Optometry and Visual Science Eye Hospital of Wenzhou Medical University Wenzhou 325027 China; ^2^ National Clinical Research Center for Ocular Diseases Eye Hospital of Wenzhou Medical University Wenzhou 325027 China; ^3^ Department of Ophthalmology Affiliated Hospital of Yanbian University Yanji 136200 China; ^4^ Department of Ophthalmology Yinzhou People's Hospital Medical School of Ningbo University Ningbo 315040 China

**Keywords:** engineered sEVs, glaucoma, miR‐29, plasma‐derived sEVs, RGCs degeneration

## Abstract

Glaucoma is a leading cause of irreversible blindness worldwide and is characterized by progressive retinal ganglion cell (RGC) degeneration and vision loss. Since irreversible neurodegeneration occurs before diagnosable, early diagnosis and effective neuroprotection are critical for glaucoma management. Small extracellular vesicles (sEVs) are demonstrated to be potential novel biomarkers and therapeutics for a variety of diseases. In this study, it is found that intravitreal injection of circulating plasma‐derived sEVs (PDEV) from glaucoma patients ameliorated retinal degeneration in chronic ocular hypertension (COH) mice. Moreover, it is found that PDEV‐miR‐29s are significantly upregulated in glaucoma patients and are associated with visual field defects in progressed glaucoma. Subsequently, in vivo and in vitro experiments are conducted to investigate the possible function of miR‐29s in RGC pathophysiology. It is showed that the overexpression of miR‐29b‐3p effectively prevents RGC degeneration in COH mice and promotes the neuronal differentiation of human induced pluripotent stem cells (hiPSCs). Interestingly, engineered sEVs with sufficient miR‐29b‐3p delivery exhibit more effective RGC protection and neuronal differentiation efficiency. Thus, elevated PDEV‐miR‐29s may imply systemic regulation to prevent RGC degeneration in glaucoma patients. This study provides new insights into PDEV‐based glaucoma diagnosis and therapeutic strategies for neurodegenerative diseases.

## Graphical abstract

Plasma‐derived small extracellular vesicles (sEVs) from glaucoma patients (Gla‐PDEV) alleviate optic nerve injury. Elevated levels of PDEV‐miR‐29s are associated with the progression of glaucoma. The delivery of miR‐29b‐3p by engineered sEVs protects retinal ganglion cells (RGCs). PDEV may be involved in the systemic regulation respond to glaucomous neurodegeneration. PDEV‐miR‐29s are potential biomarkers and promising therapeutic agents for glaucoma.

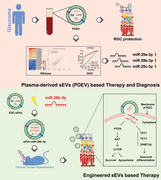

## 1 Introduction

Glaucoma is a multifactorial degenerative optic neuropathy characterized by cupping of the optic nerve head and visual field damage. It is the most frequent cause of irreversible blindness worldwide.^[^ ^1^ ^]^ Nevertheless, the underlying pathological mechanism is still poorly understood. As a neurodegenerative disease, glaucoma has long preclinical phases with progressively irreversible pathology. Diagnosis is often delayed due to the frequently asymptomatic until a relatively late stage.^[^ ^1^ ^]^ Clinical symptomatic treatments are limited and cannot effectively control the progressive loss of retinal ganglion cells (RGCs).^[^ ^2^ ^]^ Therefore, reliable noninvasive early diagnostic biomarkers and potential therapeutic targets are urgently needed.

The administration of autologous blood derivatives as a therapeutic option has acquired great prominence in ophthalmology.^[^ ^3^ ^]^ Autologous serum (AS), platelet‐rich plasma (PRP), plasma rich in growth factors and deproteinized calf blood extractives have been used in ocular and other neurodegenerative disease treatments.^[^ ^4^, ^5^, ^6^, ^7^ ^]^ As blood derivatives, small extracellular vesicles (sEVs) have great potential in the treatment of glaucoma. sEVs are 30–150 nm endogenous membrane vesicles released from all cell types to biofluids, including blood and tears.^[^ ^8^ ^]^ sEVs participate in intercellular communication locally or over long distances by transmitting bioactive molecules, such as proteins, lipids and nucleic acids.^[^ ^9^ ^]^ With the advantages of low immunogenicity and toxicity,^[^ ^10^ ^]^ high stability and biocompatibility,^[^ ^11^ ^]^ and the ability to traverse intact biological barriers, such as the blood‐brain barrier or blood‐eye barrier,^[^ ^12^ ^]^ sEVs are considered to be ideal cell‐free therapeutic nanoparticles, and the first phase I clinical trial of sEVs transplantation has been performed in delayed wound healing.^[^ ^13^ ^]^ On the other hand, sEVs molecules can remain stable for long periods and are promising noninvasive diagnostic biomarkers for complex diseases, such as progressive neurodegenerative disorders.^[^ ^14^ ^]^ Moreover, evidence suggests that the secondary immune or autoimmune responses induced by elevated intraocular pressure (IOP) occur in glaucoma^[^ ^15^, ^16^ ^]^ and systemically regulate the sEVs component derived from immune cells. This makes it possible to identify specific biomarkers from circulating plasma‐derived sEVs (PDEV) for glaucoma.

MiRNAs are endogenous noncoding RNA molecules with 20–25 nucleotides that act as important posttranscriptional regulators of gene expression by binding with their target mRNAs and play a key role in substantial biological and pathogenic processes. Importantly, miRNAs have also been identified as important regulators of neurodegenerative diseases, including AD, PD and glaucoma.^[^ ^17^ ^]^ Moreover, miRNAs have been found to be enriched in sEVs.^[^ ^18^ ^]^ Dysregulated miRNAs enriched in PDEV can be detected in association with diverse pathological conditions and have revealed diagnostic and monitoring potential in human diseases.^[^ ^19^ ^]^

In this study, we investigated the therapeutic effect of PDEV of glaucoma patients in a glaucomatous mouse model. We identified potential PDEV‐miRNA biomarkers for the diagnosis or monitoring of glaucoma progression. We further determined the effect of engineered sEVs‐miR‐29b‐3p in a glaucomatous mouse model to understand the systemic regulatory mechanisms and possible treatments.

## 2 Results

### 2.1 Circulating Plasma‐Derived sEVs (PDEV) from Glaucoma Patients Alleviate Glaucomatous Optic Neuropathy in COH Mice

**Figure 1:**
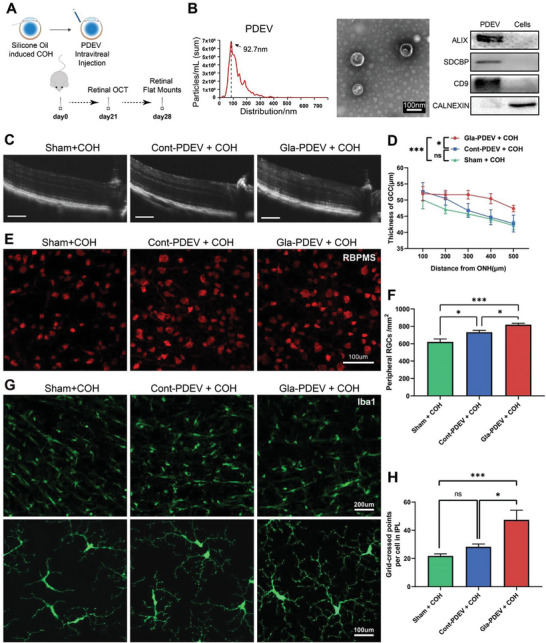
Intravitreal injection of Gla‐PDEV alleviated glaucomatous optic neuropathy in COH mice. A) Schematic illustration of the in vivo experiment. Mice were injected with silicone oil immediately followed by PDEV injection. Retinal OCT and flat‐mount imaging were conducted 3 and 4 weeks after COH. B) NTA revealed the size distribution of PDEV, TEM showed the typical morphology of PDEV, and WB confirmed the expression of specific markers (ALIX, SDCBP, CD9, and calnexin) in PDEV. C,D) Representative OCT images and quantification of retinal thickness after different treatments in COH mice (Bar = 100 µm, *n* = 9‐12). E,F) Representative flat‐mount confocal images and quantification of RGC numbers showing surviving RBPMS‐positive (red) RGCs in the peripheral retina (*n* = 8). G,H) Flat‐mount confocal images showing the distribution of GFP‐positive microglia in the 3 groups and quantification of the grid‐crossed points per microglia in the IPL (*n* = 6). (ns *p *> 0.05, *p < 0.05, ***p < 0.001).

Autologous cell/serum transplantation is a promising therapeutic approach for numerous diseases. sEVs play roles in intercellular communication through autocrine and paracrine functions and play roles in systemic regulation through endocrine mechanisms. To explore the therapeutic potential of autologous circulating PDEV transplantation in glaucoma, we treated chronic ocular hypertension (COH) mice, a model of glaucomatous optic neuropathy, with PDEV in glaucoma patients (**Figure** **1**A). We collected plasma samples from 40 primary glaucoma patients and 20 controls without optic nerve injury (Table S1 and Figure S1, Supporting Information). The isolated PDEV was confirmed to be a typical cup shape with an average particle size of 127.4 nm and 71.4−79.6% in the range of 30–200 nm, which was consistent with that of typical sEVs (Figure 1B). We also confirmed the expression of sEV‐specific markers by western blotting (ALIX, SDCBP, and CD9) and flow cytometry (FCM) (CD63 and CD81) (Figure 1B; Figure S2 and Table S2, Supporting Information).

We established a COH mouse model by anterior chamber injection of silicone oil, which resulted in angle closure with IOP elevation (Figure S2A,B, Supporting Information) and decreased thickness of the ganglion cell complex (GCC) layer and RGC numbers (Figure S3C–F, Supporting Information). Interestingly, OCT analysis revealed a significantly thicker GCC layer in Gla‐PDEV (PDEV from glaucoma patient) treated COH mice (Figure 1C,D). Whole flat‐mount retinal immunostaining also revealed significantly increased RGC survival in Gla‐PDEV treated COH mice (Figure 1E,F). Since microglia mediate inflammation in the process of RGC degeneration, we also analyzed the activation of microglia. There were more grid‐crossed points of microglia in the inner plexiform layer (IPL) in Gla‐PDEV treated mice, and there were no differences between the Sham (untreated) and Cont‐PDEV (PDEV from cataract patient) groups (Figure 1G,H). This finding may suggest that microglial activation was inhibited by Gla‐PDEV treatment.

We then conducted transcriptome sequencing of PDEV and PDEV‐treated retinas. A total of 8529 differentially expressed genes (DEGs), including 6386 upregulated and 2143 downregulated mRNAs, were identified in Gla‐PDEV compared to controls (Figure S4A, Supporting Information). GO‐BP analysis revealed biological processes enriched not only in the nervous or eye system but also in immune‐related processes, including T‐cell activation, chemokine‐mediated signaling and lymphocyte or mononuclear cell differentiation (Figure S4B, Supporting Information), which indicated the potential role of PDEV in immunoregulation. The Venn diagram shows specific and coexpressed mRNAs between Gla‐PDEV‐treated, Cont‐PDEV treated, and sham retinas (Figure S4C, Supporting Information). The heatmap also showed DEGs between the groups (Figure S4D, Supporting Information). Many biological processes were enriched in immune or inflammation as well as mitochondrial respiratory activity and response to interferon‐γ (Figure S4E,F, Supporting Information).

### 2.2 PDEV‐miRNA Signatures and Candidate Biomarkers for Glaucoma Diagnosis

**Figure 2:**
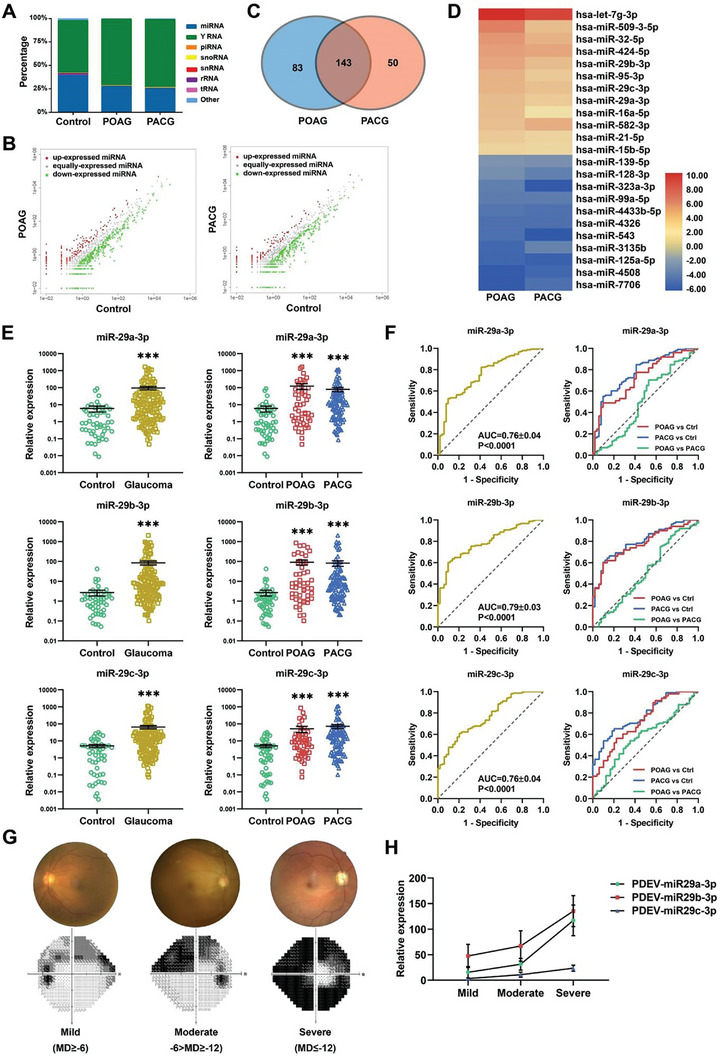
Identification and validation of candidate PDEV‐miRNAs with glaucoma specificity. A) Distribution of small RNAs detected by RNA‐seq in PDEV (*n* = 20 for each group; mt‐tRNA, transfer RNA located in the mitochondrial genome; rRNA, ribosomal RNA; piRNA, PIWI‐interacting RNA; snoRNA, small nucleolar RNA; snRNA, small nuclear RNA). B) Scatter plot showing differentially expressed PDEV‐miRNAs in glaucoma patients compared to controls (fold change = 2 as the threshold). C) A Venn diagram showing that 143 differentially expressed PDEV‐miRNAs overlapped between POAG and PACG patients. D) Twenty‐three candidate miRNAs were selected from the RNA‐seq data, including the top 12 upregulated miRNAs and the top 11 downregulated miRNAs. E) qPCR analysis of individual patient samples confirmed that miR‐29s were significantly upregulated in both POAG and PACG patients (*n* = 51 for control and POAG, *n* = 102 for PACG). F) ROC analysis showing the diagnostic performance of PDEV‐miR‐29a‐3p, miR‐29b‐3p, and miR‐29c‐3p in POAG and PACG. G,H) Glaucoma patient classification based on MD values and PDEV‐miR‐29s expression increased with disease severity. (***p < 0.001).

To profile the PDEV‐miRNAs and further identify potential biomarkers, we collected plasma samples from 283 glaucoma patients (101 patients with primary open‐angle glaucoma (POAG) and 182 patients with primary angle‐closure glaucoma (PACG)) and 101 controls. The following experiments were performed as shown in the discovery roadmap (Table S1 and Figure S1, Supporting Information). First, we pooled 20 plasma samples from each group for PDEV extraction and conducted high‐throughput small RNA sequencing. The results showed that miRNAs were enriched in PDEV (**Figure** **2**A). Compared with those in the controls, 193 and 226 miRNAs were differentially expressed in the PACG and POAG patients, respectively (fold change ≥ 2, *p* value < 0.05) (Figure 2B), among which 143 miRNAs overlapped (Figure 2C). Based on small RNA sequencing and Venn diagram analysis, 23 dysregulated miRNAs (Figure 2D) were selected for qRT‒PCR verification using pooled PDEV samples from 30 POAG patients, 60 PACG patients (30 patients with chronic angle‐closure glaucoma (CACG) and 30 patients with acute angle‐closure glaucoma (AACG)) and 30 controls. The results confirmed that 6 PDEV‐miRNAs (miR‐29a‐3p, miR‐29b‐3p, miR‐29c‐3p, miR‐21‐5p, miR‐15b‐5p, and miR‐424‐5p) were consistent with the RNA sequencing results and were significantly upregulated in glaucoma patients (POAG and PACG) (Figure S5A, Supporting Information). Then, the 6 PDEV‐miRNAs were further quantified individually in another 51 POAG patients, 102 PACG patients (51 CACG and 51 AACG) and 51 controls. The results indicated that the expression of these PDEV‐miRNAs increased significantly in primary glaucoma patients (Figure 2E; Figure S5B,C and Table S3, Supporting Information). Furthermore, the diagnostic performance of PDEV‐miR‐29s was evaluated by receiver operating characteristic (ROC) curve analysis. The expression of PDEV‐miR‐29s for glaucoma diagnosis exhibited AUC (area under the curve) values of 0.76, 0.79, and 0.76, and for POAG and PACG diagnosis, the AUC values ranged from ≈0.708 to 0.786 (Figure 2F; and Table S4, Supporting Information).

### 2.3 Correlations Between PDEV‐miRNA Levels and Clinical Indices

To further investigate the correlations between the expression of PDEV‐miRNAs and the clinical indices of glaucoma patients, an expression‐phenotype association analysis was conducted. Based on clinical examinations with Humphrey visual field, Optical Coherence Tomography and Goldmann applanation tonometry, the following parameters were obtained: mean deviation (MD), pattern standard deviation (PSD), visual field index (VFI), retinal nerve fiber layer thickness (RNFL), and intraocular pressure (IOP). The expression of PDEV‐miR‐29a‐3p, PDEV‐miR‐29b‐3p, and PDEV‐miR‐29c‐3p was associated with MD in primary glaucoma patients (*n* = 60, Pearson's correlation *coefficients (r)* = −0.37, −0.30, and −0.31; *p* = 0.003, 0.01 and 0.01, respectively) (Figure S6A, Supporting Information). To further understand the possible correlation between visual field defects and PDEV‐miR‐29 levels, we classified primary glaucoma patients as mild (MD≥−6), moderate (−6>MD≥−12), or severe (MD≤−12) based on MD values. The expression of PDEV‐miR‐29s in moderate and severe glaucoma patients was significantly greater than that in patients with mild glaucoma (Figure 2G,H), and the Pearson correlation coefficients were more significant in progressed glaucoma patients (Figure S6B,C and Table S5, Supporting Information). Further analysis revealed weak correlations between PDEV‐miR‐29a‐3p and RNFL thickness and between PDEV‐miR‐29b‐3p and the VFI in glaucoma patients (Figure S6D, Supporting Information, red dots). These results suggested that PDEV‐miR‐29s may be a useful biomarker for evaluating RGC degeneration in primary glaucoma patients.

### 2.4 Verification of the Specificity of PDEV‐miR‐29s

**Figure 3:**
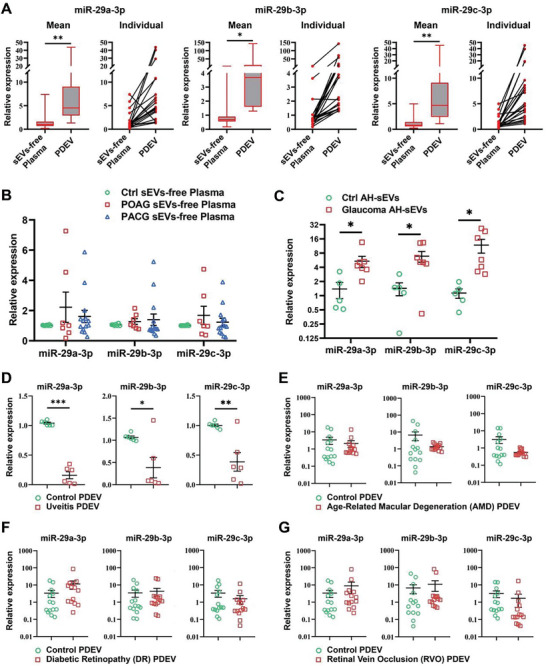
Expression of miR‐29s in sEVs‐free plasma and AH‐sEVs and detection of PDEV‐miR‐29s in other eye disease types. A) The expression of miR‐29s in PDEV was significantly greater than that in sEVs‐free plasma at both the average and individual levels (*n* = 21). B) The expression of miR‐29s in sEVs‐free plasma did not differ between the glaucoma patients and controls (*n* = 7). C) The expression of miR‐29s in AH‐sEVs from glaucoma patients was significantly increased (*n* = 7). D–G) The expression of PDEV‐miR‐29s in the uveitis (D), AMD (E), DR (F), and RVO (G) groups was compared with that in the control group (*n* = 6 for the uveitis group, *n* = 14 for the AMD, DR, and RVO groups). (*p < 0.05, **p < 0.01, ***p < 0.001).

To verify the specificity and feasibility of PDEV‐miR‐29s levels in glaucoma patients, we collected both PDEV and sEV‐free plasma samples and quantified the expression of miR‐29s in the same patients. The results showed that the mean and individual expression of miR‐29s in PDEV were significantly greater than those in sEV‐free plasma (**Figure** **3**A). We also determined that the miR‐29s levels in the sEV‐free plasma were not significantly different between the glaucoma and control groups (Figure 3B). Furthermore, we observed that the miR‐29s levels in the aqueous humor (AH) of glaucoma patients were significantly greater than those in the controls, which is consistent with the PDEV trends (Figure 3C). Additionally, to investigate the specificity of elevated PDEV‐miR‐29s in glaucoma, we obtained PDEV from patients with other ocular diseases, including uveitis, AMD, DR, and RVO. The expression of PDEV‐miR‐29s was significantly decreased in patients with uveitis but was not different in patients with other ocular fundus diseases (Figure 3D–G). These results suggested that elevated PDEV‐miR‐29s can be a clinically available noninvasive body fluid biomarker for the diagnosis and prognosis of glaucoma patients.

### 2.5 Administration of miR‐29b‐3p Mitigates RGC Degeneration in COH Mice

**Figure 4:**
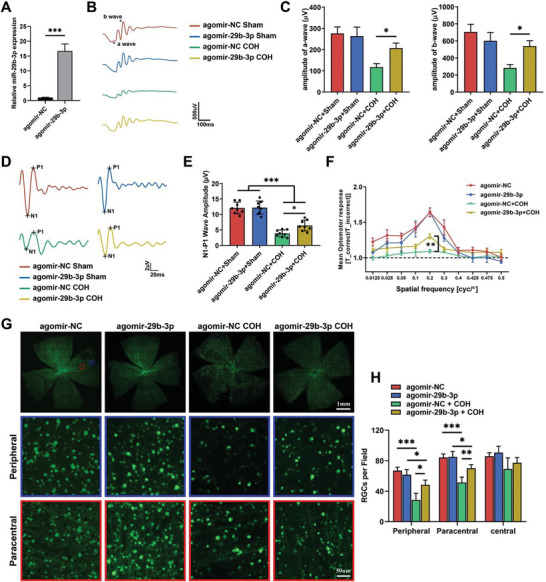
Overexpression of miR‐29b‐3p in the retina prevents retinal degeneration in COH mice. A) Intravitreal injection of agomir‐29b‐3p significantly increased miR‐29b‐3p levels in the mouse retina. The a‐ and b‐wave amplitudes under scotopic conditions B,C) (*n* = 12) and the N1‐P1 amplitude of FVEP D,E) (*n* = 10) significantly increased in miR‐29b‐3p‐overexpressing mice 4 weeks after COH. F) Visual function under a spatial frequency of 0.2 cyc/d was significantly improved in miR‐29b‐3p‐overexpressing mice 4 weeks after COH (*n* = 6). G,H) RGC survival in the peripheral and paracentral areas was greater in whole flat‐mounted retinas from miR‐29b‐3p‐overexpressing mice than in those from control mice (*n* = 5). (*p < 0.05, **p < 0.01, ***p < 0.001).

Numerous studies have suggested systemic immune and inflammatory responses to neuronal injury.^[^ ^20^, ^21^, ^22^ ^]^ However, the mechanism by which PDEV‐miR‐29s are elevated in glaucoma patients remains to be studied. To determine whether PDEV‐miR‐29s play a systemic regulatory role in glaucoma progression, we conducted rescue experiments through intravitreal miR‐29b‐3p treatment of COH mice. The expression of miR‐29b‐3p was effectively upregulated by intravitreal injection of agomir‐29b‐3p (**Figure** **4**A). ERG, FVEP, and qOMR analyses revealed that agomiR‐29b‐3p treatment partially restored RGC function in COH mice (Figure 4B–F). Moreover, whole flat‐mount retina analysis revealed that miR‐29b‐3p administration increased RGC survival in the peripheral and paracentral retinas of COH mice (Figure 4G,H).

**Figure 5:**
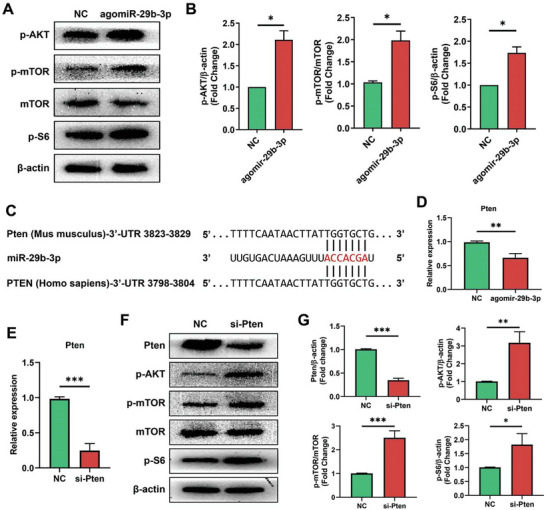
miR‐29b‐3p regulates the Pten‐dependent AKT/mTOR pathway. A) Western blots showing high levels of phosphorylated AKT, mTOR and S6 in retinas overexpressing miR‐29b‐3p. B) Quantification of the data in A, *n* = 3. C) Alignment between the binding sites of miR‐29b‐3p and Pten. D) qPCR showed a significant decrease in the level of the Pten mRNA in miR‐29b‐3p‐overexpressing cells. E) qPCR showing efficient knockdown of Pten mRNA in retinas after Pten‐specific siRNA transfection. F) Western blots showing decreased Pten protein levels in Pten‐knockdown cells, which resulted in increased levels of phosphorylated AKT, mTOR, and S6. G) Quantification of the data in F, *n* = 3. (*p < 0.05, **p < 0.01, ***p < 0.001).

To investigate the neuroprotective mechanism of miR‐29b‐3p, we analyzed the activation of the AKT/mTOR pathway by western blotting in miR‐29b‐3p‐treated retinas. The results showed that the phosphorylation levels of AKT, mTOR, and S6 were significantly elevated (**Figure** **5**A,B). Pten is a negative regulator of the AKT/mTOR pathway and a target gene of miR‐29b‐3p.^[^ ^23^ ^]^ The seed sequence of miR‐29b‐3p was complementary to the 3′ untranslated region (3′ UTR) of Pten in both human and mouse sequences (Figure 5C). The mRNA level of Pten decreased when miR‐29b‐3p was overexpressed in Neuro2A cells (Figure 5D). Next, we transfected Neuro2A cells with a Pten‐specific siRNA, which resulted in successful knockdown of Pten mRNA (Figure 5E). Similar to miR‐29b‐3p overexpression, Pten knockdown also increased the levels of phosphorylated AKT, mTOR and S6 (Figure 5F,G). These results indicated that miR‐29b‐3p promotes AKT/mTOR pathway activation by inhibiting its target gene Pten.

### 2.6 MiR‐29b‐3p Promotes Neuronal Differentiation of Neural Stem Cells

**Figure 6:**
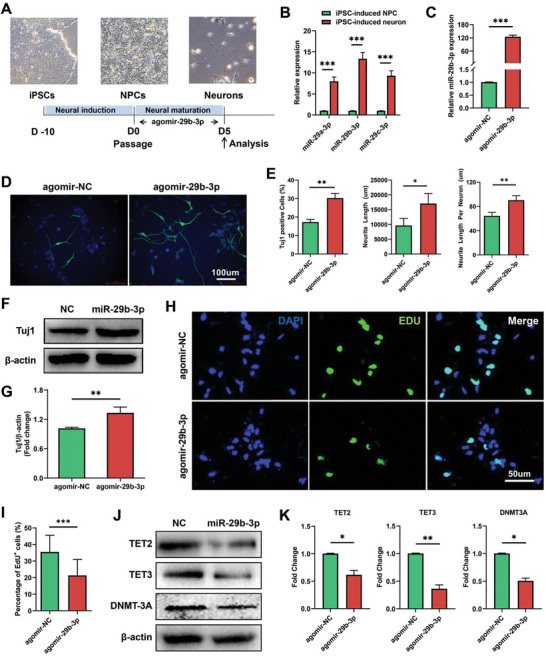
Overexpression of miR‐29b‐3p promotes the neuronal differentiation of ESC‐induced NPCs. A) Schematic diagram of neuronal differentiation. B) The expression of miR‐29s was significantly increased during differentiation. C) Agomir‐29b‐3p transfection efficiently increased miR‐29b‐3p levels in NPCs. D,E) miR‐29b‐3p significantly increased the number of Tuj1‐positive cells, total length, and average length of neurites after 5 days of differentiation. F,G) miR‐29b‐3p significantly increased the protein level of Tuj1 in differentiated cells. H,I) miR‐29b‐3p inhibited the proliferation of NPCs, as detected by an EdU assay. J,K) The expression of the DNA methylation‐related genes TET2, TET3, and DNMT3A significantly decreased in miR‐29b‐3p‐overexpressing cells. (*p < 0.05, **p < 0.01, ***p < 0.001).

It has been demonstrated that miRNAs play important roles in the nervous system. Our previous study showed that the expression of miR‐29s increased during retinal development.^[^ ^24^ ^]^ To further investigate the role of miR‐29s in neuronal differentiation, hiPSCs were directly differentiated in monolayers to NPCs, in turn to early neurons (**Figure** **6**A). Directional neuronal differentiation can simulate the process of in vivo neuronal development to a certain extent. The expression of miR‐29s was significantly increased during neuronal differentiation (Figure 6B). NPCs were transfected with miR‐29b‐3p, and the overexpression of miR‐29b‐3p was confirmed by qPCR 48 hours after transfection (Figure 6C). On day 5 of terminal differentiation, overexpression of miR‐29b‐3p significantly increased the number of Tuj1‐positive neuronal cells and neurite length (Figure 6D,E), and the protein level of Tuj1 was also significantly increased (Figure 6F,G). The cell proliferation assay showed that the number of EdU‐positive cells was significantly decreased in the miR‐29b‐3p‐overexpressing group (Figure 6H,I). To further investigate the mechanism by which miR‐29b‐3p promotes neuronal differentiation, we also analyzed the expression of DNA methylation‐related proteins, which are known factors involved in neuronal development.^[^ ^25^, ^26^ ^]^ The results showed that miR‐29b‐3p administration significantly decreased the protein levels of TET2, TET3, and DNMT3A (Figure 6J,K). DNA methylations are known to be important events that regulate cell differentiation during development. Numerous studies have indicated that genes related to stemness are methylated and that lineage‐specific genes undergo demethylation.^[^ ^27^, ^28^ ^]^ These results suggest that miR‐29b‐3p promoted early neuronal differentiation through DNA methylation regulation.

### 2.7 Engineered sEVs‐miR‐29b‐3p Promote Retinal Neuroprotection

**Figure 7:**
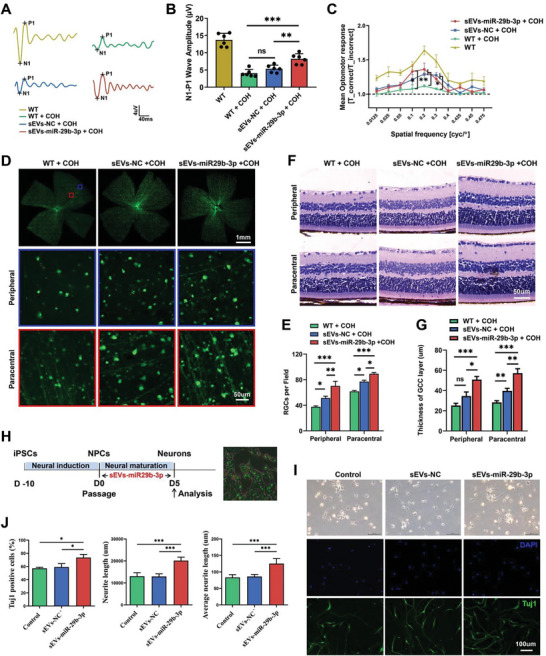
sEVs‐mediated delivery of miR‐29b‐3p mitigated RGC degeneration in COH mice and enhanced in vitro neuronal differentiation efficiency. A,B) The N1‒P1 amplitude of FVEP significantly increased in sEVs‐miR‐29b‐3p‐injected mice 3 weeks after COH (*n* = 6). C) The visual function of sEVs‐miR‐29b‐3p‐injected mice was significantly improved (*n* = 6). D,E) RGC survival in the peripheral and paracentral areas was increased in whole flat‐mounted retinas of sEVS‐miR‐29b‐3p‐injected mice (*n* = 7). F,G) The thickness of the ganglion cell complex (GCC) layer was restored in sEVs‐miR‐29b‐3p‐injected retinas (*n* = 6). H) Schematic diagram of sEVsthe effect of sEVs‐miR‐29b‐3p treatment on neuronal differentiation. I,J) Increased numbers of Tuj1‐positive cells and increased total length and average length of neuronal neurites were observed in the sEVS‐miR29b‐3p‐treated group. (ns *p *> 0.05, *p < 0.05, **p < 0.01, ***p < 0.001).

To investigate the function of miR‐29b‐3p delivered by sEVs in RGC protection, we conducted the following experiments. Embryonic stem cell (ESC)‐derived sEVs were isolated from the culture supernatant by ultracentrifugation. TEM images revealed small round vesicles, and the particles of the sEVs were ≈50–200 nm (108 nm on average) in diameter (Figure S7A,B, Supporting Information). Western blotting was also performed using the known sEV‐positive markers TSG101, CD9, and CD81 and the sEV‐negative marker calnexin (Figure S7C, Supporting Information). To visualize the mobility of sEVs in the retina, a PKH67 green fluorescent cell linker was used to label the sEVs, which were then intravitreally injected into the mouse model. Whole flat‐mounted retinas showed efficient internalization of sEVs by retinal cells (Figure S7D, Supporting Information). Engineered sEVs‐miR‐29b‐3p were constructed by electroporation, and the expression levels of miR‐29b‐3p increased more than 500‐fold compared to those in the NC group (Figure S7E, Supporting Information). We observed efficient upregulation of miR‐29b‐3p by intravitreal injection of sEVs‐miR‐29b‐3p, indicating successful small RNA delivery by sEVs in the retina (Figure S7F, Supporting Information). Three weeks after sEVs‐miR‐29b‐3p treatment in COH mice, we observed a partially restored amplitude of FVEP (**Figure** **7**A,B) and improved visual function (Figure 7C). Histopathological analysis revealed increased RGC survival following sEVs‐miR‐29b‐3p administration (Figure 7D,E), and increased retinal thickness, especially in the GCC layer, was observed in sEVs‐miR‐29b‐3p‐treated COH mice (Figure 7F,G). Interestingly, ESC‐sEVs also played a role in neuroprotection in this study, whereas sEVs‐miR‐29b‐3p had a more significant effect. To further investigate the function of sEVs‐delivered miR‐29b‐3p in neuronal differentiation in vitro, engineered sEVs‐miR‐29b‐3p was administered to NPCs, followed by 5 days of terminal differentiation (Figure 7H). We observed increased numbers of Tuj1‐positive neurons and total neurite length as well as average neurite length per neuron in the sEVs‐miR‐29b‐3p‐treated group but not in the ESC‐sEVs‐only group (Figure 7I,J). This finding suggested that miR‐29b‐3p plays an important role in neuronal differentiation with sufficient delivery by sEVs.

### 2.8 Transcriptomic Analysis of sEVs‐miR‐29b‐3p‐Treated Retinas from COH Mice

**Figure 8:**
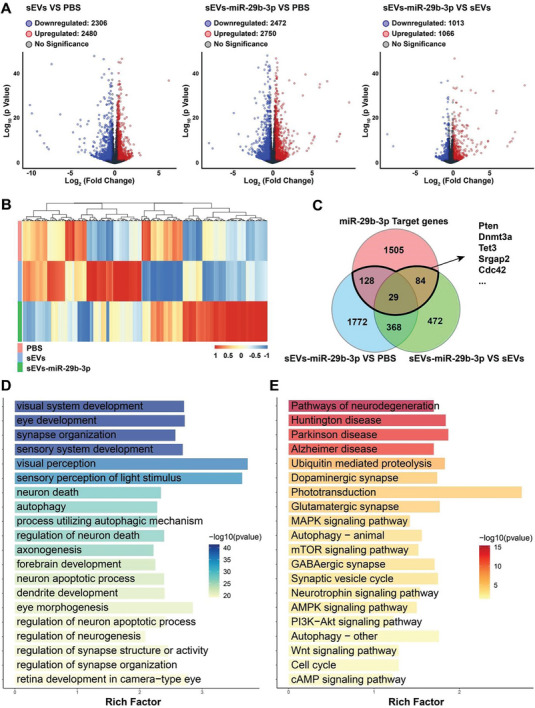
Transcriptomic analysis of sEVs‐miR‐29b‐3p‐treated retinas from COH mice. A) Volcano plots showing the number of downregulated and upregulated genes among the 3 groups. B) Heatmap of DEGs in the control, sEVs‐treated, and sEVs‐miR‐29b‐3p‐treated groups. C) Venn diagram for the analysis of genes that were differentially expressed in the sEVs‐miR‐29b‐3p group and verified miR‐29b‐3p targets. D,E) GO and KEGG pathway enrichment analyses of DEGs between the sEVs‐miR‐29b‐3p‐treated group and the PBS‐treated group.

To further understand the molecular mechanisms involved in the retina, transcriptome sequencing was performed on control, sEVs‐treated and sEVs‐miR‐29b‐3p‐treated retinas from COH mice. Volcano plots revealed 2480, 2750, and 1066 upregulated genes and 2306, 2472, and 1013 downregulated genes in the sEVs versus control, sEVs‐miR‐29b‐3p versus sEVs, and sEVs‐miR‐29b‐3p versus control groups, respectively (**Figure** **8**A). The heatmap showed different gene expression patterns between the groups (Figure 8B). Then, we performed a bioinformatics analysis of the verified target genes of miR‐29b‐3p with downregulated genes in the sEVs‐miR‐29b‐3p versus sEVs and sEVs‐miR‐29b‐3p versus control groups. Pten, Dnmt3a, Tet3, Srgap2, and Cdc42, which participate in neuroprotection/differentiation and DNA methylation, were involved in the intersections shown in the Venn diagram (Figure 8C). The differentially expressed genes in sEVs‐miR‐29b‐3p retinas were enriched in several functional processes and pathways, including synapse organization, neuron death, autophagy, neurodegeneration, and the MAPK, PI3K‐Akt‐mTOR, and Wnt pathways, according to GO and KEGG enrichment analyses (Figure 8D,E). These findings also confirmed the role of miR‐29b‐3p in retinal degeneration and the underlying mechanisms involved.

## 3 Discussion

sEVs are secreted from all live cells containing biomolecules, such as nucleic acids, lipids and proteins, and can deliver these cargoes into nearby cells as well as over long distances via blood.^[^ ^29^ ^]^ sEVs contribute to cell‐cell communication through paracrine and endocrine delivery of molecules, which play important roles in diseases. sEVs contain autoantigens that may regulate immune responses in the circulatory system.^[^ ^30^ ^]^ Herein, we investigated whether there is a systemic feedback regulatory mechanism in the PDEV of glaucoma patients.

Glaucoma is a common neurodegenerative eye disorder characterized by RGC degeneration and irreversible blindness. It is mainly a symptomatic treatment in the clinic, such as lowering intraocular pressure, but there are still some patients who eventually become blind. Autologous blood derivatives therapy is frequently used in the clinic, such as AS or PRP for ocular disease, such as dry eye syndrome.^[^ ^4^, ^31^, ^32^ ^]^ We are curious whether PDEV from glaucoma patients plays a neuroprotective role in glaucomatous optic neuropathy. Our novel study showed that Gla‐PDEV mitigates RGC degeneration in preclinical mice. We speculate that the PDEV of glaucoma patients is similar to that of preconditioned situation. We believe that PDEV may be safer and more powerful in promoting clinical application than EVs from allogenetically cultured cells with unexpected immunogen proteins in cultured media.

PDEV are stable as they avoid phagocytosis by circulating monocytes and macrophages,^[^ ^33^ ^]^ which may support their promising value in disease diagnosis.^[^ ^34^ ^]^ PDEV‐miRNAs are attractive blood‐based biomarkers for neurodegenerative disorders such as Alzheimer's disease (AD) and Parkinson's disease (PD).^[^ ^35^, ^36^ ^]^ Increasing evidence has indicated that up to half of glaucoma patients may lose their visual field before clinical diagnosis because there are no obvious symptoms in the early stage.^[^ ^37^ ^]^ Thus, the discovery of biofluid‐based glaucoma biomarkers is urgently needed for the clinical diagnosis and early intervention of glaucoma. Previous studies have attempted to discover biological molecules based on the origin of pathological retinal neurons, but they are generally not recognized because it is difficult to find trace amounts of molecules in circulating blood.^[^ ^38^ ^]^

Our novel study confirmed that six PDEV‐miRNAs (miR‐29a‐3p, miR‐29b‐3p, miR‐29c‐3p, miR‐21‐5p, miR‐15b‐5p, and miR‐424‐5p) were significantly upregulated in primary glaucoma patients. Interestingly, several recent studies determined that sEVs‐miR‐21‐5p levels in plasma/serum significantly increased in many diseases, such as depressive disorder, pancreatitis, non‐small cell lung cancer, diabetic cardiomyopathy, and glioblastoma.^[^ ^39^, ^40^, ^41^, ^42^, ^43^ ^]^ These results are consistent with our findings, and we speculate that PDEV‐miR‐21‐5p can be considered to have alarmin‐like properties for disease warming. Numerous studies have suggested that the associations between glaucoma and degenerative diseases of the central nervous system, such as AD, PD, anxiety, and depression, are associated with glaucoma.^[^ ^44^, ^45^ ^]^ Moreover, it has demonstrated that glaucomatous neurodegeneration is accompanied by secondary immune or autoimmune responses,^[^ ^16^, ^46^ ^]^ resulting in changes in immunomodulation, which leads to alterations in the PDEV‐miRNA composition. Glaucoma is not only a local ocular disease but also a systemic regulatory mechanism involved in disease progression. Thus, the PDEV of glaucoma patients may originate from organs, including the retina and brain, while others originate from blood cells or immune cells. We hypothesize that the contribution of elevated PDEV‐miR29s is intended for systemic feedback regulation in response to RGC degeneration.

Our ROC analysis revealed that PDEV‐miR‐29s have great sensitivity and specificity for detecting glaucoma. The MD and VFI are major parameters of visual field tests that can reflect glaucoma features and are commonly used clinically to evaluate glaucoma progression. Elevated PDEV‐miR‐29s were positively correlated with the degree of visual field defect (MD). Mckiernan reported a urinary exosomal 3‐gene expression assay for clinical use to predict high‐grade prostate cancer.^[^ ^47^, ^48^ ^]^ This kit, developed by ExoDx, has been approved by the Food and Drug Administration (FDA). Therefore, the combination of PDEV‐miR29s may be a promising biomarker for noninvasive glaucoma diagnosis, but prospective and multicenter studies may be needed in the future.

The PDEV‐miR‐29s were significantly higher than that in sEVs‐free plasma, and no difference was observed in sEVs‐free plasma between glaucoma and control (Figure 3A,B), suggesting the enrichment of miRNAs in PDEV. Aqueous humor (AH) may more directly reflect intraocular conditions, including retinal disorders. Our study showed that the levels of miR‐29s in the PDEV and AH exhibited the same upward trends in individual glaucoma patients (Figure 3C). Notably, a recent study also reported that miRNAs were elevated in both the AH and plasma of glaucoma patients.^[^ ^38^ ^]^ One of the limitations of this study was that we used age‐related cataract patients instead of healthy controls due to ethical and sampling restrictions. However, most previous studies have used age‐related cataracts as a control group in ophthalmic research.^[^ ^49^, ^50^ ^]^ To further verify the specificity of elevated PDEV‐miR‐29s in primary glaucoma patients, we also quantified the expression of PDEV‐miR‐29s in other retinal diseases, such as AMD, DR and RVO, as well as in uveitis, an ocular inflammatory disease. We observed significant differences only in uveitis. Similarly, a previous study reported that miR‐29s were downregulated in HLA‐B27‐associated acute anterior uveitis.^[^ ^51^ ^]^ Based on these findings, it is highly possible that PDEV‐miRNA detection could be a useful approach for noninvasive biomarker discovery in primary glaucoma.^[^ ^52^ ^]^

Herein, we investigated whether there is a systemic feedback regulatory mechanism resulting in significantly elevated PDEV‐miR‐29s in glaucoma patients. MiRNAs regulate abundant gene expression, playing a crucial role in diverse cellular activities, and dysregulation of their expression is involved in the pathogenesis of human diseases.^[^ ^53^ ^]^ Studies have shown that the downregulation of miR‐29b in the brain is involved in AD and HD development.^[^ ^54^ ^]^ Our recent study reported that miR‐29a has antiangiogenic effects in preclinical ocular models.^[^ ^55^ ^]^ We observed that miR‐29s were decreased in the retinas of COH mice (data not shown), which was similar to the results of a previous study.^[^ ^56^ ^]^ Therefore, to verify the function of miR‐29s in glaucomatous optic neuropathy, we intravitreally treated COH mice with miR‐29b‐3p and found increased RGC survival with alleviated visual loss (Figure 4). AKT/mTOR signaling is the most well‐known neuroprotective pathway in several nerve injuries, including RGC degeneration.^[^ ^57^ ^]^ Our study also confirmed that the AKT/mTOR pathway was markedly activated by miR‐29b‐3p administration in the retinas of COH mice.

Neurogenesis is an important physiological process of the nervous system. Understanding the mechanism of neurogenesis is highly important for the treatment of neurodegenerative diseases.^[^ ^58^ ^]^ MiRNAs are important molecules that regulate neurogenesis and participate in the development of various nervous systems.^[^ ^59^ ^]^ Studies have reported that miR‐29a regulates neuronal differentiation^[^ ^60^ ^]^ and that miR‐29b regulates mouse embryonic neurogenesis.^[^ ^61^ ^]^ Our previous study demonstrated that miR‐29 levels increase continuously during retinal development.^[^ ^24^ ^]^ In this study, we also detected low levels of miR‐29s in iPSC‐induced NPCs and increased miR‐29s expression during neuronal differentiation. In addition, we found that miR‐29b‐3p overexpression inhibited NPC proliferation and promoted neuronal differentiation (Figure 6). Epigenetic modification, such as DNA methylation, is important for embryonic development and cell differentiation processes. Tet methylcytosine dioxygenase (TET1, TET2, and TET3) and DNA methyltransferase (DNMT3A, DNMT3B) are known enzymes that mediate DNA methylation.^[^ ^62^ ^]^ DNMTs and TETs are the targets of miR‐29s; for example, miR‐29b promotes somatic cell reprogramming by targeting DNMT3a/b and TET1.^[^ ^63^ ^]^ Our results demonstrated that miR‐29b‐3p was upregulated during neuronal differentiation and that TET2, TET3, and DNMT3A were suppressed after miR‐29b‐3p overexpression (Figure 6). Thus, miR‐29b‐3p regulates DNA methylation by targeting TET2/3 and DNMT3A, resulting in efficient neuronal differentiation. These results provide guidance for understanding the biological function of miR‐29b‐3p in neuroretinal development.

Recently, growing evidence has revealed encouraging findings of the therapeutic potential of native and bioengineered sEVs for multiple diseases due to their low levels of immunogenicity and toxicity, the use of lipid membranes for ideal fusion with target cells, blood‐brain barrier breakdown ability, and functional molecular delivery.^[^ ^64^ ^]^ First, clinical platelet‐derived sEVs were delivered to patients with delayed wound healing.^[^ ^13^ ^]^ MSC‐derived EVs have been the most commonly used biomaterials for disease therapies in recent studies, even for the treatment of RGC degeneration through miRNA delivery.^[^ ^65^ ^]^ Another study reported that hESC‐derived EVs alleviate retinal degeneration by promoting the retrodifferentiation of Müller cells into RPCs.^[^ ^66^ ^]^ Additionally, several studies have shown efficient miRNA delivery to the central nervous system (CNS) when sEVs are loaded with exogenous miR‐124, miR‐210 and miR‐126, and sEVs‐miRNAs mitigate neurodegeneration in brain disease.^[^ ^67^, ^68^, ^69^ ^]^ To further investigate the function of bioengineered sEVs in RGC degeneration, we loaded hESC‐sEVs with miR‐29b‐3p to generate sEVs‐miR‐29b‐3p and treated COH mouse retinas. Our results showed that sEVs‐miR‐29b‐3p exhibited more therapeutic effects with more efficient miR‐29b‐3p delivery than direct miR‐29b‐3p administration in the COH model. Furthermore, sEVs are thought to regulate neurogenesis following the alleviation of neuronal damage.^[^ ^70^, ^71^ ^]^ However, our results demonstrated that sEVs‐miR‐29b‐3p promoted neuronal differentiation but not in the ESC‐sEVs treated group, suggesting that miR‐29b‐3p plays a major role in this process. A previous study reported the therapeutic effects of transplanted exosomes in AD models through miR‐29b delivery,^[^ ^72^ ^]^ which also reinforces our confidence. We next performed transcriptomic analysis of sEVs‐miR‐29b‐3p‐treated retinas, and GO and KEGG enrichment analyses revealed potential neuroprotective pathways. Interestingly, we identified 266 miR‐29b‐3p target genes, such as Pten, Dnmt3a, Tet3, Srgap2, and Cdc42, among the differentially expressed genes in ESC‐sEVs and sEVs‐miR‐29b‐3p‐treated retinas. These genes are associated with DNA methylation and neuroprotection,^[^ ^73^ ^]^ and some of them were validated by our qPCR and WB analyses. Taken together, based on the elevated circulating plasma sEVs‐miR‐29 levels in glaucoma patients, we speculated that the systemic feedback regulatory mechanism may be involved in glaucoma progression, which, through miR‐29 delivery, leads to compensatory protection against RGC degeneration.

In conclusion, our novel study demonstrated that identifying PDEV‐miRNAs could be a useful strategy for biofluid‐based noninvasive biomarker discovery. The underlying mechanism of elevated PDEV‐miR‐29s may involve systemic feedback regulation to prevent RGC degeneration in glaucoma patients. Our data suggest that PDEV‐miR‐29s are useful biomarkers for glaucoma diagnosis and that autologous PDEV or bioengineered sEVs‐miR‐29s are promising therapeutic approaches for neurodegenerative diseases.

## 4 Experimental Section

### Patient Characteristics and Collection of Circulating Plasma Samples

A total of 283 glaucoma patients (including 101 with primary open‐angle glaucoma (POAG) and 182 with primary angle‐closure glaucoma (PACG)), 6 with uveitis, 14 with AMD, 14 with DR and 14 with RVO, and 101 with cataracts) were enrolled in this study, which was approved by The Ethics Committee of the Eye Hospital of Wenzhou Medical University on Biomedical Research Involving Human Subjects (2022‐091‐K‐69). All patients were diagnosed with primary glaucoma with 1) a history of highest intraocular pressure (IOP) greater than 21 mmHg; 2) angular abnormalities on gonioscopy; 3) glaucomatous optic disc damage (vertical cup‐to‐disc ratio >0.6 and/or interocular asymmetry of cup‐to‐disc ratio >0.2); 4) corresponding visual field defects; or 5) no other fundus diseases, ocular traumas, or systemic diseases. All participants were questioned about age, sex, history of ocular disease, and systemic clinical symptoms. The VF and IOP were measured bilaterally, and a slit‐lamp examination and OCT were performed. The general characteristics of the glaucoma patients are shown in Table S3 (Supporting Information). The mean age ranged from 49.73 to 64.06 years among the groups. The mean IOP, such as decreased visual field index (VFI), mean deviation (MD), and pattern standard deviation (PSD), in the glaucoma group was significantly greater than that in the control group with RNFL thickness and visual impairment. Peripheral blood samples from glaucoma patients and controls were collected with vacutainer EDTA blood collection tubes (367 863, BD, Franklin Lakes, NJ). Cell‐free plasma was separated from blood samples within 4 h after collection. The plasma samples were then stored at −80 °C for further processing. A consensus was reached before the laboratory and clinical information was obtained.

### Animals and Experimental Mouse Models

All animal studies were conducted according to protocols approved by the Institutional Animal Care and Use Committee of Wenzhou Medical University (wydw2022‐0663) and were in accordance with the ARVO Statement for the Use of Animals in Vision Research. All mice were purchased from Vital River Laboratories (Beijing, China), fed standard laboratory chow and maintained at 21–23 °C and 55% humidity with a 12 h light/dark photoperiod. The chronic ocular hypertension (COH) model was induced by silicone oil injection into the anterior chamber, modified from a previous description.^[^ ^74^ ^]^ Male mice were anesthetized via peritoneal injection of 30 mg kg^−1^ sodium pentobarbital (P3761, Sigma, Darmstadt, Germany). A 32G needle was used to tunnel through the superotemporal side sclera close to the limbus without injuring the lens or iris. Approximately 2 µl of silicone oil (Viscosity 1000 mPa.s, S28005, Yuanye Biotech, Shanghai, China) was injected into the anterior chamber using a sterile microsyringe (Hamilton, Reno, Nevada) following entry into the sclera and the pupil. To prevent possible infections, ofloxacin eye ointment (Sinqi Pharmaceutical, Shenyang, China) was applied to the cornea every day after surgery. Mice with corneal opacity caused by band‐shaped degeneration or neovascularization were excluded from further analysis.

### sEVs Isolation and Quantification

PDEV were isolated from circulating plasma samples using a combination of centrifugation and the sEVs isolation reagent method. One milliliter of plasma was centrifuged at 2,000 × g for 10 min and then at 10,000 × g for 10 min to remove dead cells and cell debris. PDEV were harvested by 30 min of incubation with sEV isolation reagent (C10110, RiboBio, Guangzhou, China) followed by 20 min of centrifugation at 15,000 g. The PDEV pellet was washed before RNA extraction, thus limiting potential contamination by sEV‐free RNAs.

For miRNA loading, sEVs from cultured cells were isolated by ultracentrifugation. Cell culture supernatants were collected and centrifuged at 300 × g for 10 min, 2,000 × g for 10 min, 10,000 × g for 30 min and 100,000 × g for 70 min. Finally, the pellet was resuspended in PBS for miRNA loading.

sEVs were quantified as follows: size distribution and quantification were measured via ZetaView NTA (PMX 110, Particle Metrix, Meerbusch, Germany); morphology was checked through transmission electron microscopy (TEM) (H‐7650, Hitachi Ltd., Tokyo, Japan); and the expression of enriched proteins (CD63, CD81, Alix and TSG101) and the negative organelle marker calnexin were analyzed by Western blot or flow cytometry (Accuri C6 plus, BD, Piscataway, NJ). For loading ESC‐sEVs with miRNAs, equal masses of sEVs and agomir were mixed gently in PBS, and electroporation was performed with 1,000 V/cm, 10 ms and 1 pulse square wave (Gemini, BTX, Holliston, MA) and incubated at 37 °C for 30 min before and after electroporation to improve the loading efficiency.

For information on RNA extraction and sequencing, quantitative real‐time polymerase chain reaction (qRT‒PCR), GO and KEGG analysis, cell culture and differentiation, Western blot, immunostaining and EdU analysis, quantitative optomotor response (qOMR), electroretinography, and statistical analyses, please refer to the Supporting Information, Experimental Section.

## Conflict of Interest

The authors declare no conflict of interest.

## Author Contributions

T.L., W.Z., and J.W. contributed equally to this work and are co‐first authors. T.L., Q.L., W.W., and Z.C. conceived and designed the study; T.L., W.Z., J.W. B.L., and J.Z. carried out experiments and statistical analysis; Q.G., J.P., M.L. H.Q., and Q.H. assisted with the data analysis; A.F., Q.Z., X.G., R.C., and Y.L. collected patient samples; T.L., W.Z., B.L., and Z.C. wrote and edited the manuscript; and Q.L., W.W., and Z.C. administered and supervised all the experiments and analyses. The authorship order was assigned based on the degree of contribution. All the authors have read and approved the final manuscript.

## Supporting information

Supporting Information

## Data Availability

The data that support the findings of this study are available from the corresponding author upon reasonable request.
