# Correction to “Circulating Small Extracellular Vesicles Involved in Systemic Regulation Respond to RGC Degeneration in Glaucoma”

**DOI:** 10.1002/advs.202502324

**Published:** 2025-04-01

Li T, Zhang WM, Wang J, Liu BJ, Gao Q, Zhang J, Qian HD, Pan JY, Liu M, Huang Q, Fang AW, Zhang Q, Gong XH, Cui RZ, Liang YB, Lu QK, Wu WC, Chi ZL. Circulating Small Extracellular Vesicles Involved in Systemic Regulation Respond to RGC Degeneration in Glaucoma. *Adv Sci (Weinh)*. 2024 Aug;11(32):e2309307.

https://doi.org/10.1002/advs.202309307

1. **On Page 2**: In the Results Section 2.1, second paragraph, the original sentence “We established a COH mouse model by anterior chamber injection of silicone oil, which resulted in angle closure with IOP elevation (Figure **S2A, B**, Supporting Information)” should be amended to: “We established a COH mouse model by anterior chamber injection of silicone oil, which resulted in angle closure with IOP elevation (Figure **S3A, B**, Supporting Information).”

2. **Figure 7I, on Page 11**: We found an inadvertent substitution in Figure 7I, where fluorescence images of the sEVs‐miR‐29b‐3p group were erroneously selected during panel preparation. The corrected Figure 7I (provided below) now accurately represents each experimental group. As the quantification of Figure7I was based on 5 correct images from each group, the results remain unchanged. The overall findings and conclusions are not affected as well.


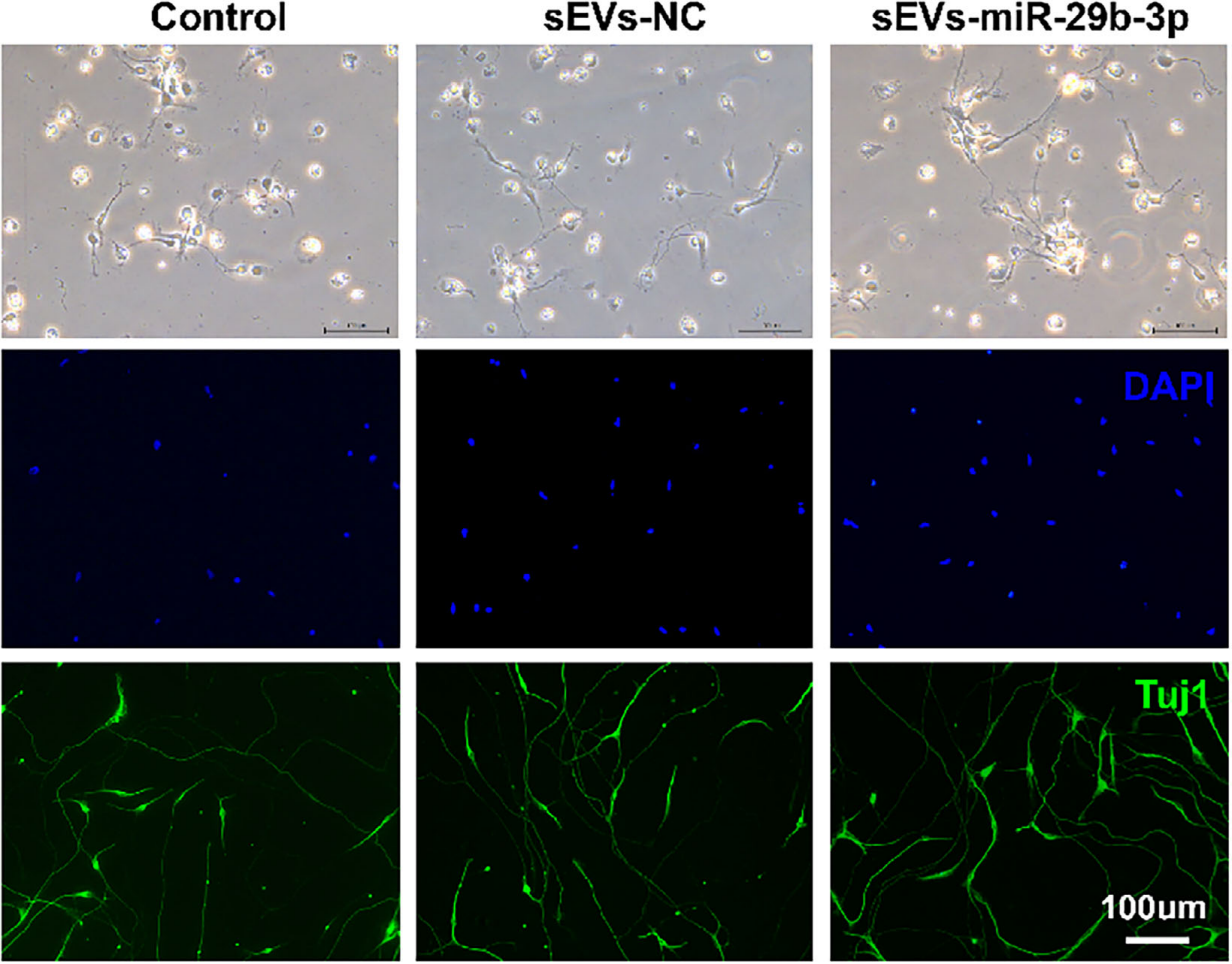


We apologize for these errors.

