# Supplementary material for: Circulating Small Extracellular Vesicles Involved in Systemic Regulation Respond to RGC Degeneration in Glaucoma
Source: Adv Sci (Weinh). 2024 Jun 25;11(32):2309307. doi: 10.1002/advs.202309307 (PMC11348076; doi:10.1002/advs.202309307)
Supplement: Supplementary file 1 — Supporting Information [file ADVS-11-2309307-s001.docx]

**Supporting Information**

**Circulating small extracellular vesicles involved in systemic regulation respond to RGC degeneration in glaucoma**

Tong Li^1#^, Wen-Meng Zhang^1#^, Jie Wang^1#^, Bai-Jing Liu^1^, Qiao Gao^1^, Jing Zhang^1^, Hai-Dong Qian^1^, Jun-Yi Pan^1^, Ming Liu^1^, Qing Huang^1^, Ai-Wu Fang^2^, Qi Zhang^2^, Xian-Hui Gong^2^, Ren-Zhe Cui^3^, Yuan-Bo Liang^1, 2^, Qin-Kang Lu^4*^, Wen-Can Wu^1, 2*^, and Zai-Long Chi^1, 2*^

^1^State Key Laboratory of Ophthalmology, Optometry and Visual Science, Eye Hospital of Wenzhou Medical University, Wenzhou, 325027, China.

^2^National Clinical Research Center for Ocular Diseases, Eye Hospital of Wenzhou Medical University, Wenzhou, 325027, China.

^3^Department of Ophthalmology, Affiliated Hospital of Yanbian University, Yanji, 136200, China;

^4^Department of Ophthalmology, Yinzhou People's Hospital, Medical School of Ningbo University, Ningbo, 315040, China.

*Correspondence to:

Zai-Long Chi, MD, PhD

Professor of Laboratory of Neurovascular Biology

State Key Laboratory of Ophthalmology, Optometry and Visual Science

Eye Hospital of Wenzhou Medical University

270 West Xueyuan Road

Wenzhou, Zhejiang 325027, P. R. China

Tel: +8618968835450

Email: [zailong.chi@eye.ac.cn](mailto:zailong.chi@eye.ac.cn)

Wen-Can Wu, MD, PhD

National Clinical Research Center for Ocular Diseases

Eye Hospital of Wenzhou Medical University

270 West Xueyuan Road

Wenzhou, Zhejiang 325027, P R. China

Tel: +8613676759736

Email: [wuwencan118@163.com](mailto:wuwencan118@163.com)

Qin-Kang Lu MD, PhD

Department of Ophthalmology

Yinzhou People's Hospital, Medical School of Ningbo University

Ningbo, Zhejiang, 315040, P. R. China.

Tel: +8613777975266

E-mail: [luqinkang@163.com](mailto:luqinkang@163.com)

^#^TL, WZ and JW contributed equally to this work and are co-first authors.

**Supplemental Text**

**Experimental Section**

**RNA extraction and sequencing.** Total RNA was extracted from PDEV or retinas using the Total RNA Kit I (Omega Biotek, Norcross, GA) according to the manufacturer’s protocol. The purity and concentration were quantified using a Multiskan GO Microplate Spectrophotometer (Thermo Fisher Scientific, Vantaa, Finland). For small RNA, libraries were constructed using the QIAseq miRNA Library Kit (Qiagen, Frederick, MD), followed by reverse transcription and polymerase chain reaction amplification. The polymerase chain reaction products derived from the 18 to 30 nucleotide RNA molecules were purified by electrophoresis and sequenced using the Illumina HiSeq 2500 platform (Illumina, San Diego, CA). For mRNA, libraries were constructed using the SMARTer Stranded Total RNA-Seq Kit (Takara Bio Inc., Shiga, Japan). After the library passed the quality check, high-throughput sequencing was conducted using an Illumina NovaSeq 6000 (Illumina, San Diego, CA). The end reading of the 150 bp pair was generated.

**Quantitative real-time polymerase chain reaction (qRT‒PCR).** The expression level of each miRNA was quantified in triplicate using the miDETECT A Track miRNA qRT‒PCR Starter Kit (C10712, RiboBio, Guangzhou, China) on QuantStudio™ 3 (Thermo Fisher Scientific, Waltham, MA). miDETECT A Track miRNA qRT‒PCR Primer (RiboBio, Guangzhou, China) was used to amplify the miRNAs. The PCR conditions were as follows: 95 °C for 10 min and 40 cycles of 95 °C for 2 s, 60 °C for 20 s, and 70 °C for 10 s. The miRNA expression levels were analyzed and quantified using the 2-△△Cq method. All Ct values >35 cycles were considered undetectable. The *C. elegans* miR-39 spike-in control (Qiagen, Duesseldorf, Germany) was added before reverse transcription. qPCR primers (forward and reverse) for each miRNA were designed by RiboBio (Guangzhou, China).

**GO and KEGG analysis.** Gene Ontology (GO) enrichment analysis was based on comparisons between DEGs and genetic backgrounds in the Gene Ontology database (http://www.geneontology.org). The *p* value denotes the significance of GO term enrichment. Kyoto Encyclopedia of Genes and Genomes (KEGG) pathway enrichment analysis is a functional analysis in which genes are mapped to KEGG pathways (http://www.genome.jp/kegg/). Lower *p* values indicate greater significance. The recommended *p* value cutoff is 0.05.

**Cell culture and differentiation.** hESCs (human embryonic stem cells H9, Stem Cell Bank/Stem Cell Core Facility, SIBCB, CAS, Shanghai, China) and hiPSCs (human induced pluripotent stem cells, SiDanSai Biotech, Shanghai, China) were cultured in E8 medium (A1517001, Gibco, Rockville, MD). hiPSC-NPCs were cultured in DMEM/F12 1:1 Neurobasal A (10888022 Gibco, Carlsbad, CA) supplemented with 2% B27 (12587010, Gibco, Rockville, MD), 1% N2 (17502048, Gibco, Rockville, MD), 1% NEAA (11140050, Gibco, Rockville, MD), 1% GlutaMAX (35050061, Gibco, Rockville, MD), 10 ng/ml bFGF (10014-HNAE, Sino Biological Inc., Beijing, China), and 10 ng/mL EGF (10605-HNAE, Sino Biological Inc., Beijing, China). The cells were cultured in Matrigel (356234, Corning)-coated dishes at 37 °C with 5% CO2 in a humidified atmosphere. For neural differentiation, NPCs transfected with agomir or incubated with sEVs were seeded on POL (P4957, Sigma)/laminin (L2020, Sigma)-coated 6-well plates at 10^5^ cells per well. The cells were cultured in DMEM/F12 1:1 Neurobasal A supplemented with 2% B27 (50X), 1% N2 (100X), 1% NEAA, 1% GlutaMAX, 64 µg/mL L-amino acids (A8960, Sigma, St. Louis, MO) and 10 µM DAPT (Selleck, Houston, Texas).

**Western blotting.** Cells or sEVs were lysed using RIPA buffer supplemented with the protease inhibitor PMSF. Then, the cells were placed on ice for 30 min and centrifuged at 17,850 rpm at 4 °C. The protein concentration in the supernatant was determined by a BCA protein analysis kit (2325, Thermo Fisher Scientific). The proteins were separated by electrophoresis and transferred to PVDF membranes. The membrane was blocked in 2.5% skim milk at room temperature for 2 hours, and primary antibodies, including anti-RBPMS (1830-rbpms, Phosphosolutions), anti-Iba1 (019-19741, Wako), anti-Tuj1 (ab78078, Abcam), anti-TET2 (ab94580, Abcam), anti-TET3 (NBP2-20602, Novus), anti-DNMT3a (3598, CST), anti-p-AKT (4060, CST), anti-p-mTOR (5536, CST), anti-mTOR (2972, CST), anti-p-S6 (5364, CST), anti-PTEN (9188, CST), anti-β-actin (AF0003, Beyotime), anti-TSG101 (ab125011, Abcam), anti-BDCBP (D223488-0025, BBI), anti-CD9 (60232-1-IG, Proteintech), Alix (12422-1-AP, Proteintech), CD81 (PTM-6220, PTM BIO), anti-CD63 (ab68418, Abcam) and Calnexin (10427-2-AP, Proteintech) at a 1:1,000 ratio were incubated at 4 °C overnight. After washing in Tris-buffered saline containing Tween 20 (P5416, Sigma, Darmstadt, Germany), the membrane was incubated with the secondary antibody at a 1:2,000 ratio at room temperature for one hour. The protein bands were visualized in a gel imaging system (FL1500, Invitrogen, Singapore), and the data were analyzed by ImageJ.

**Immunostaining and EdU analysis.** Whole flat-mounted retinas or cells cultured on coverslips were fixed with 4% PFA and blocked with 3% BSA at room temperature (RT) for 30 min. Primary antibodies, including anti-Tuj1 (ab78078, Abcam) and anti-RBPMS (15187-1-AP, Proteintech), were incubated at a 1:500 ratio at 4 °C overnight. The primary antibodies were revealed with Alexa 488-conjugated donkey anti-rabbit at RT for 1 h. EdU staining was performed using an EdU Kit (C10310-3, RiboBio, Guangzhou, China) according to the manufacturer’s protocol.

**Quantitative optomotor response (qOMR).** The mice were placed on a platform surrounded by four screens to create a box (PHENOSYS qOMR, Berlin, Germany). The screens displayed a moving grid creating a virtual cylinder at 100% contrast at 10 different given frequencies (0.012, 0.025, 0.05, 0.1, 0.2, 0.3, 0.4, 0.425, 0.475 and 0.5 c/d (cycles/degree)). Body movements in mice were quantified using video tracking at the top of the box by determining the speed of movement of the center of mass and the angular body velocity derived through a linear regression of the whole body. Clockwise rotation of the grid and tracking represent the left eye, while counterclockwise rotation and tracking represent the right eye.

**Electroretinography.** For electroretinography (ERG) analysis, mice were dark-adapted overnight and anesthetized. After 5 min of dilation, the mice were stimulated by flashing light varying in intensity from -5.0 to 35 log scotopic Candela Second/m2 (cd·s/m2) in a Ganzfeld dome (Q400, Roland Consult, Wiesbaden, Germany). The stimulus light intensity was attenuated with neutral density filters (Kodak, Rochester, NY), and luminance was calibrated with an IL-1700 integrating radiometer/photometer (International Light, Newburyport, MA).

**Statistical analyses.** Animal and cell experiments were performed at least three times (n≥3), and the specific sample size was provided in the legends of figures. The statistical data are presented as means ± SEM. Student’s t test (two groups) or one-way ANOVA (three groups and more) followed by Tukey’s post hoc test was perfomed by GraphPad Prism 8 software. *p* < 0.05 was considered to indicate statistical significance. The area under the receiver operating characteristic curve (AUC) was used to evaluate the diagnostic value of miRNA expression for glaucoma. The best cutoff value was selected based on the principle of minimizing the sensitivity-specificity difference and maximizing the discriminating power of the tests. The Kruskal–Wallis test was used for age, highest IOP, and RNFL thickness comparisons. Chi-square with Bonferroni correction was used for sex comparisons. Statistical differences were analyzed by Student’s t test or Tukey’s post hoc test after one-way ANOVA for multiple comparisons of mean values. *p* < 0.05 was considered statistically significant.

**Supplemental Tables**

**Table S1.** Clinical data of patients for PDEV intravitreal injection and specific PDEV-miRNA screening.

Table S2 Analysis of the PDEV-specific markers CD63 and CD81 by flow cytometry

Table S3 Relative expression levels of the six PDEV-miRNAs in different types of glaucoma

Table S4 The diagnostic power of PDEV-miR-29s for different types of glaucoma

Table S5. Correlation of PDEV-miR-29s with MD≥-6 (mild glaucoma) or MD≤-6 (moderate and severe glaucoma)

**Supplemental Figures**


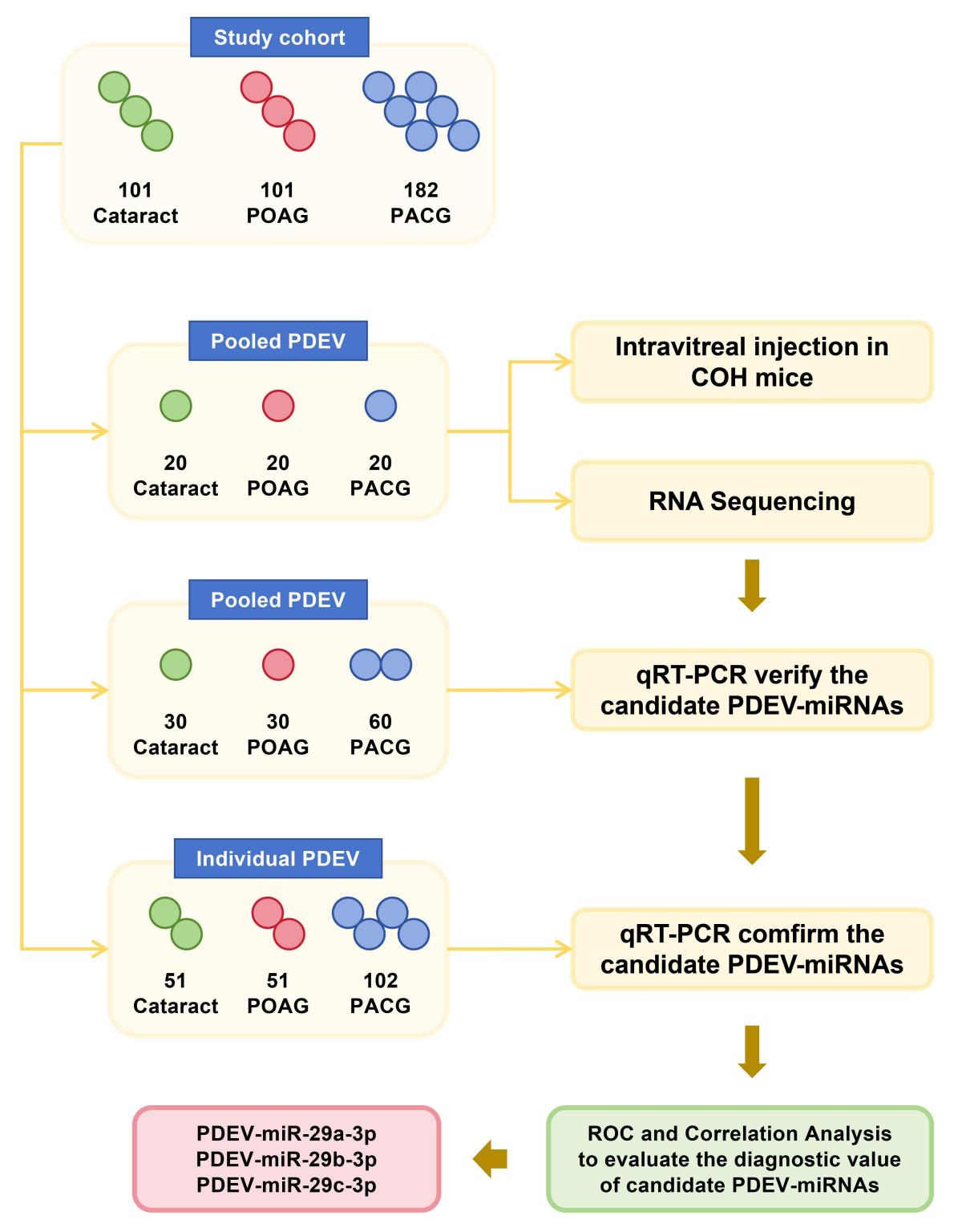


Figure S1. Study roadmap for PDEV treatment in COH mice and glaucoma specific PDEV-miRNA screening


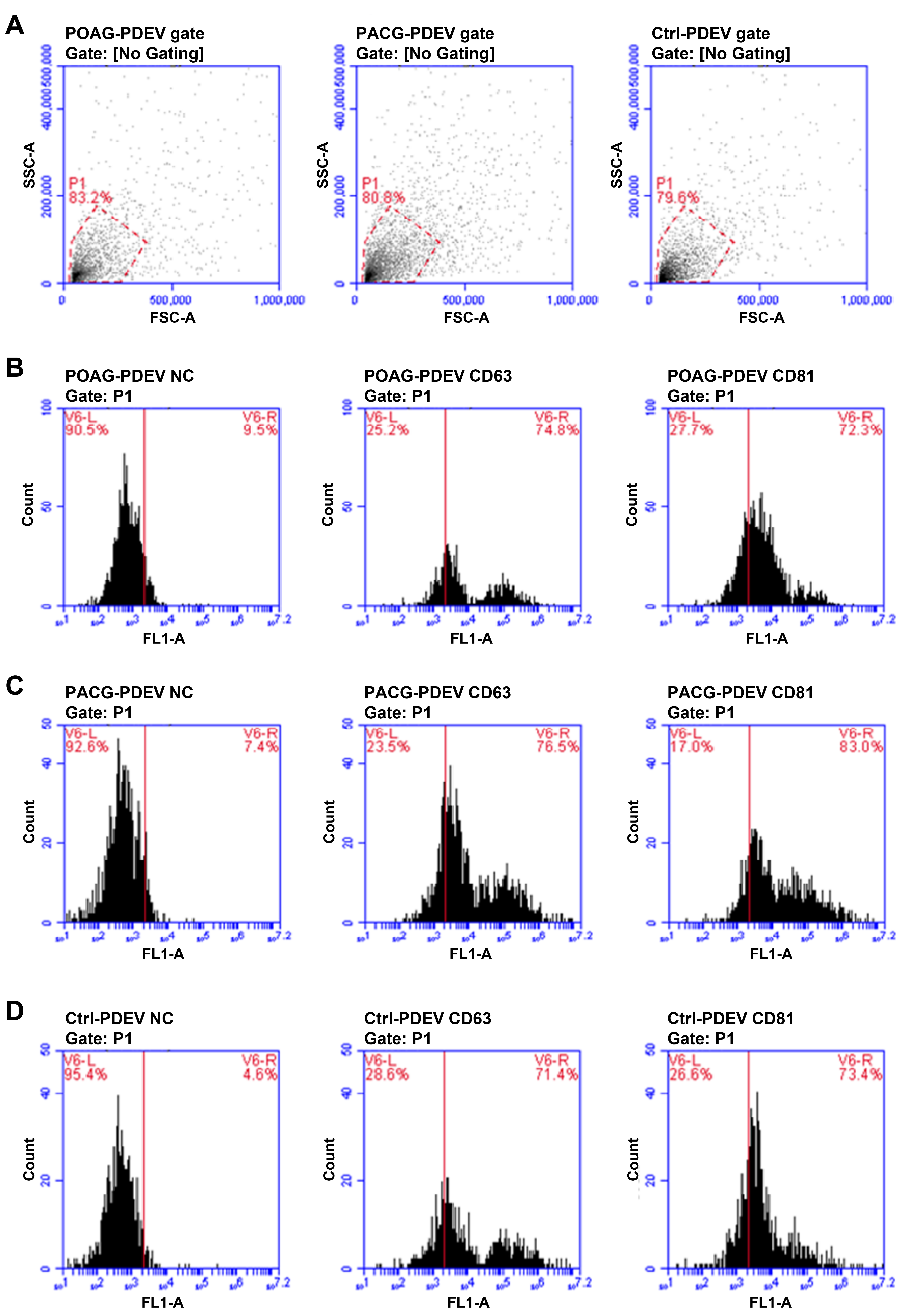


Figure S2. Flow cytometry (FCM) analysis of PDEV from POAG, PACG and Ctrl plasma with specific exosomal markers CD63 and CD81.


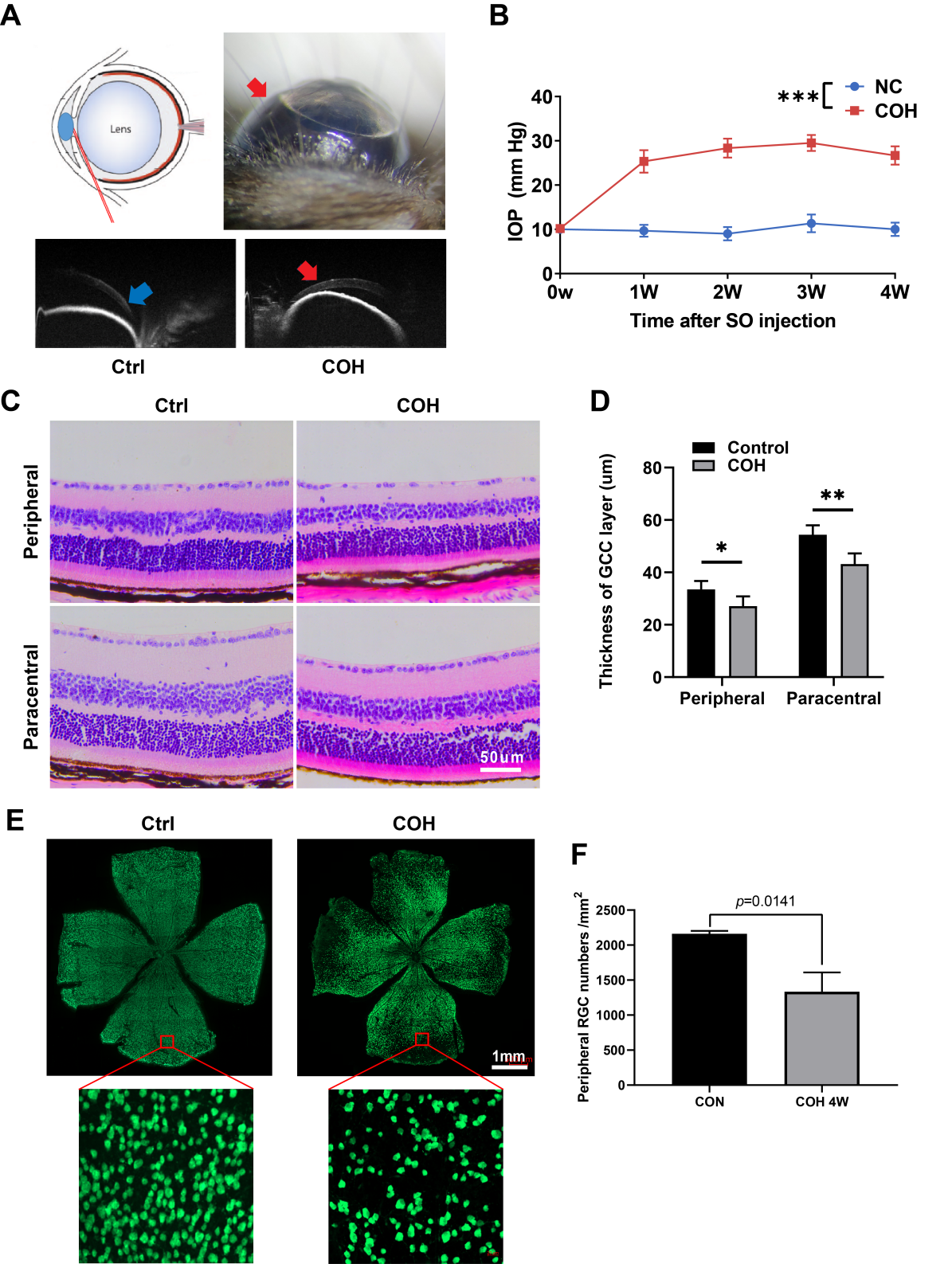


Figure S3. Establishment of the chronic ocular hypertension (COH) mouse model. (A) Silicon oil (SO) intracameral injection resulted in pupillary block by the SO droplet and anterior chamber angle closure due to the high pressure from the posterior segment induced by the accumulation of aqueous humor. (B) IOP measurements at different time points after SO injection. (C, D) HE staining analysis of the morphological changes and thickness of the GCC layer 4 weeks after COH in the mouse retina. (C) Representative confocal images showing RBPMS-positive (green) RGCs in the whole flat-mounted retina and the quantification of surviving RGCs 4 weeks after COH. The data are shown as the mean±SEM, n =6.


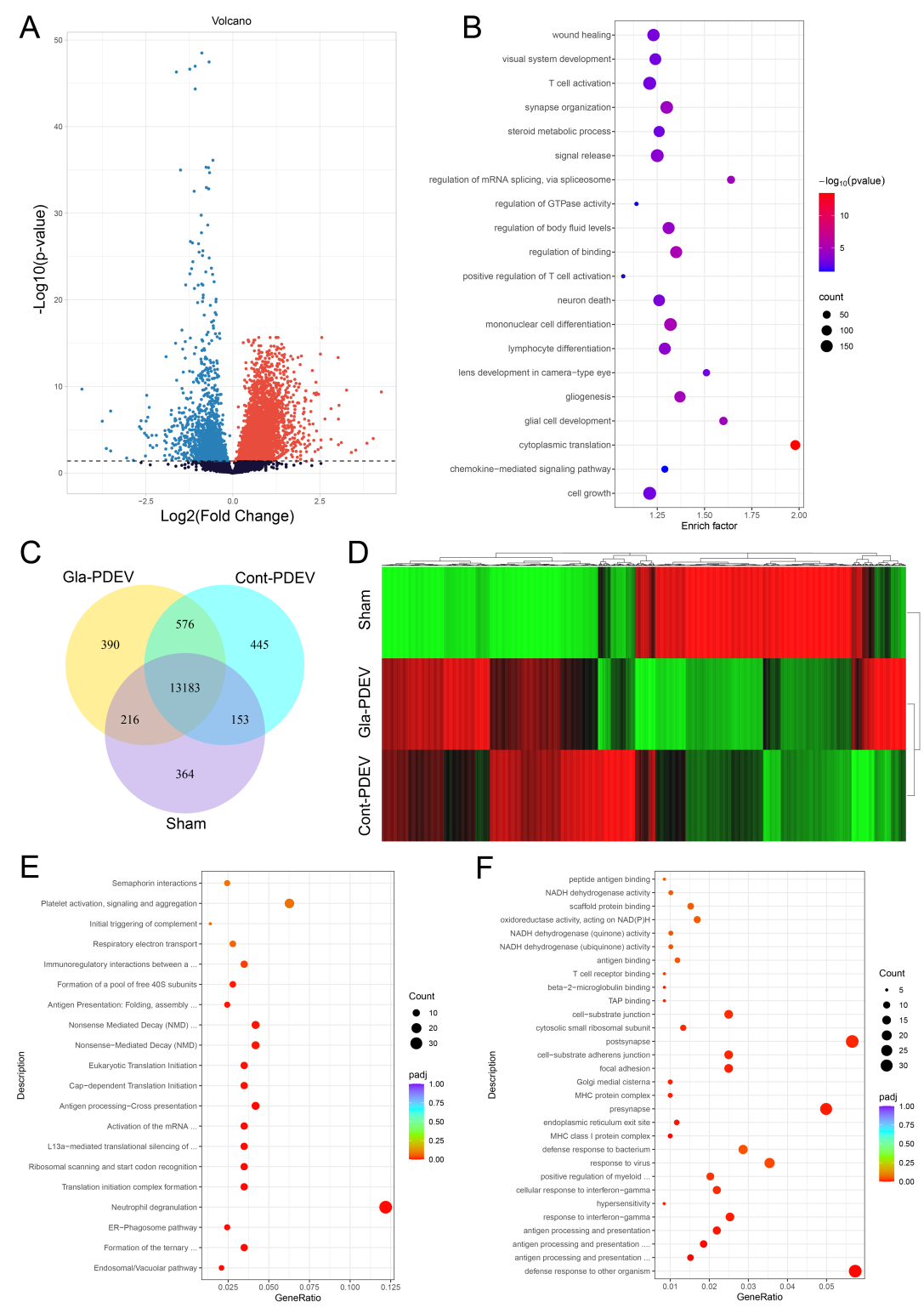


Figure S4. Transcriptomic analysis of PDEV- and PDEV-treated retinas from glaucoma patients in COH mice. (A, B) The differentially expressed mRNAs in the PDEV of glaucoma patients compared with the PDEV of control patients and the subsequent GO-BP analysis. (C) The numbers of unique and shared mRNAs in the 3 groups. (D) Heatmap of differentially expressed genes in the 3 groups. (E) GO analysis of differentially expressed genes between Gla-PDEV- and Cont-PDEV-treated retinas from COH mice. (F) Reactome pathway analysis of differentially expressed genes between Gla-PDEV- and Cont-PDEV-treated retinas from COH mice


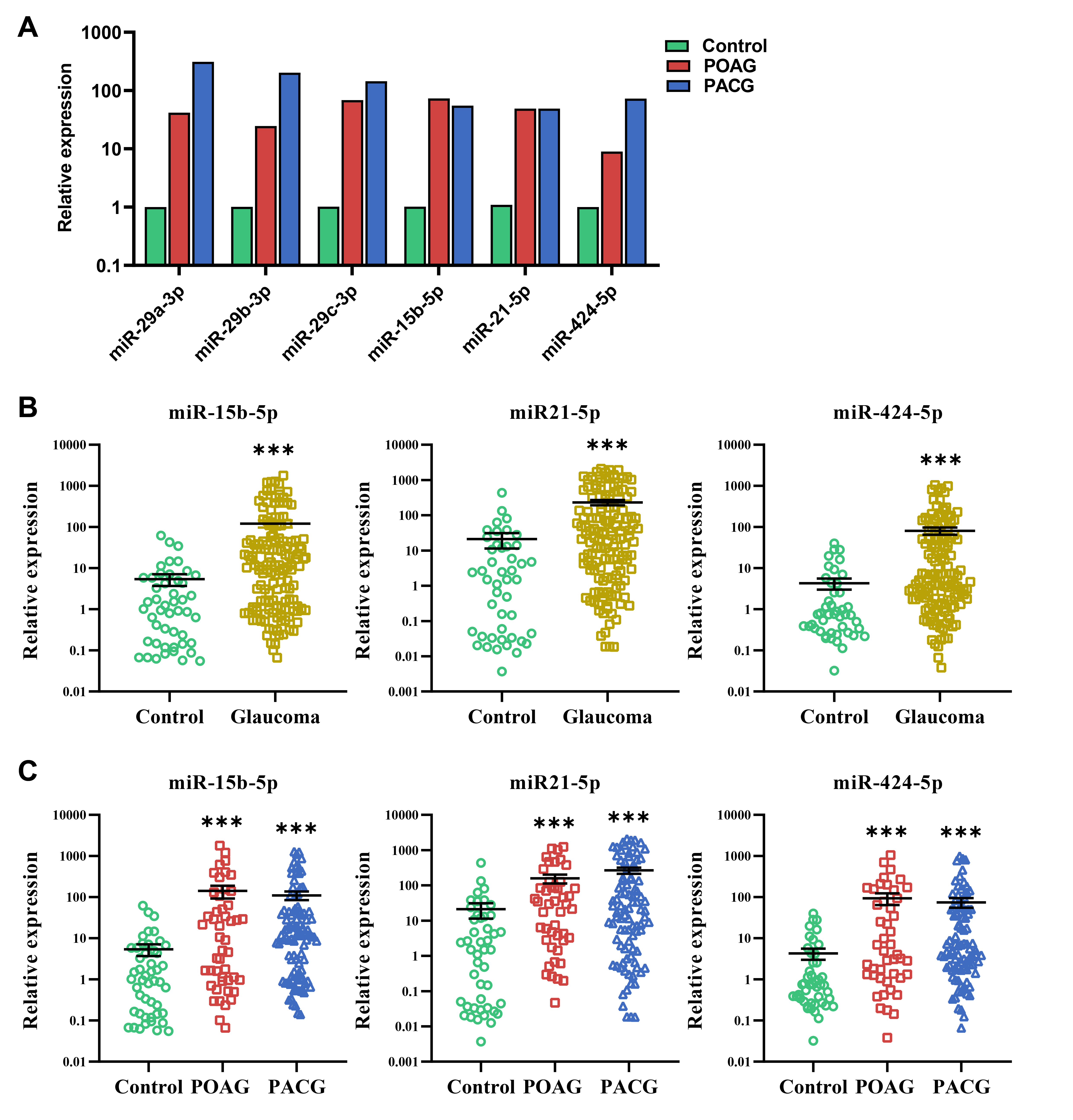


Figure S5. Identification and validation of candidate PDEV-miRNAs with glaucoma specificity. (A) qPCR of pooled samples from 30 patients verified that 6 of 23 miRNAs were consistent with the sequencing results. (B, C) qPCR analysis of individual patient samples confirmed that miR-15b-5p, miR-21-5p and miR-424-5p were significantly upregulated in both POAG and PACG patients. (n=51 for Ctrl and POAG, n=102 for PACG, ***p*<0.01, ****p*<0.001)


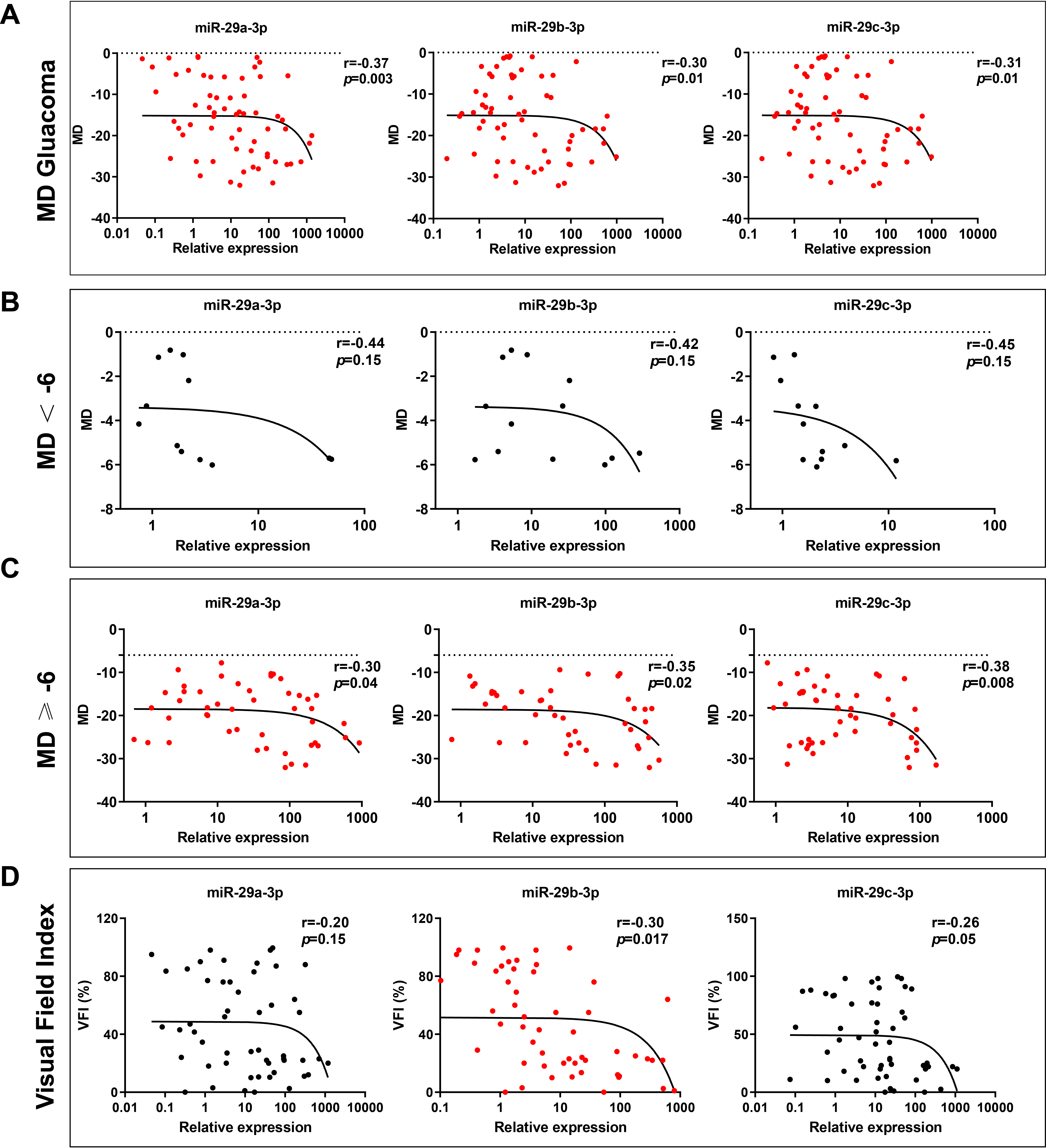


Figure S6. Correlations between the expression of PDEV-miR-29s and the mean deviation (MD) and visual field index (VFI). (A) PDEV-miR-29a-3p, miR-29b-3p and miR-29c-3p were associated with MD in glaucoma patients. (B, C) PDEV-miR-29s were more significant in guiding the visual progression of patients with moderate and severe glaucoma (MD<-6) than in patients with mild glaucoma (MD≥-6). (D) Correlation between PDEV-miR-29s and the VFI. (Every point represents the average of two diseased eyes or just one diseased eye. r = Pearson’s correlation coefficient. )


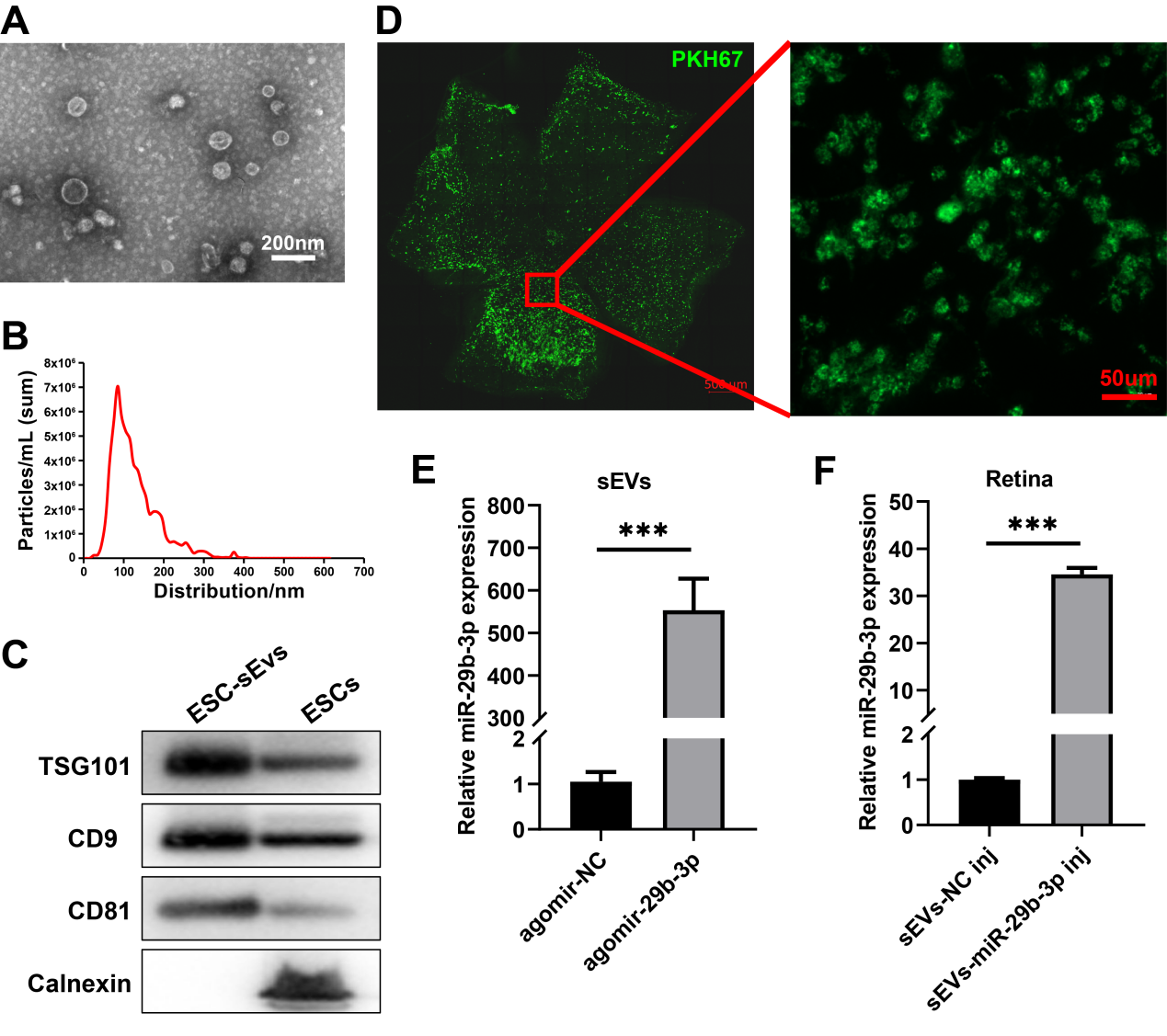


Figure S7. Identification of hESC-derived sEVs loaded with miR-29b-3p and internalization by retina cells. (A) Typical cup-shaped morphology of ESC-sEVs. (B) The major particle size of ESC-sEVs is approximately 50-200 nm. (C) ESC-sEVs express the specific markers TSG101, CD9 and CD81 but not calnexin. (D) PKH67-labeled sEVs were efficiently internalized by retinal cells after intravitreal injection. (E, F) sEVs electroporated with the same weight of agomir-29b-3p resulted in a 550-fold increase in miR-29b-3p in sEVs and a 35-fold increase in miR-29b-3p in the intravitreally injected retina.
